# Hepatic arterial infusion chemotherapy (HAlC) versus sorafenib for hepatocellular carcinoma (HCC) in Barcelona Clinic Liver Cancer (BCLC) B/C: A systematic review and meta-analysis

**DOI:** 10.1371/journal.pone.0342495

**Published:** 2026-02-18

**Authors:** Zhengqiang Chen, Yaotian Fan, Yuting Luo, Xiwen Ye, Zhen You

**Affiliations:** 1 Department of General Surgery, Division of Biliary Tract Surgery, West China Hospital, Sichuan University, Chengdu, China; 2 General Practice Medicine Center, West China Hospital, Sichuan University, Chengdu, Sichuan, China; Yonsei University College of Medicine, KOREA, REPUBLIC OF

## Abstract

**Introduction and objectives:**

Despite both hepatic arterial infusion chemotherapy (HAIC) and sorafenib being efficacious for hepatocellular carcinoma (HCC), choosing between them for Barcelona Clinic Liver Cancer (BCLC) stages B/C patients remains controversial. This meta-analysis aims to compare their therapeutic outcomes and prognoses in such patients.

**Methods:**

Pubmed, EMBASE, and Web of Science databases were searched. The primary outcome of this meta-analysis is Overall Survival (OS), while secondary outcomes include Progression-Free Survival (PFS), tumor response rate, and the incidence of adverse events. The analysis has included a total of 18 studies, comprising 3008 patients in aggregate.

**Results:**

The analysis revealed a combined Hazard Ratio (HR) for OS of 0.57 (95% CI 0.38–0.86) and for PFS of 0.46 (95% CI 0.38–0.57). Subgroup analysis by different HAIC regimens: FOLFOX-based HAIC regimens 0.28 (95% CI: 0.16–0.50), FP regimen 0.68 (95% CI: 0.25–1.87), New-FP regimen 0.60 (95% CI: 0.47–0.77), cisplatin-based HAIC 0.63 (95% CI: 0.47–0.85). The pooled ORs were: Complete Response (CR) 3.88 (95% CI 1.56–9.65), Partial Response (PR)4.72(95% CI 2.44–9.13), Stable Disease (SD) 0.83 (95% CI 0.45–1.53), Progressive Disease (PD) 0.35 (95% CI0.25–0.48, Objective Response Rate (ORR) 5.32 (95% CI 2.54–11.13), Disease Control Rate (DCR) 2.03 (95% CI 1.05–3.92). For adverse events (AEs), the overall incidence Odds Ratios (OR) was 0.53 (95% CI 0.06–4.82) and for grade 3−4 events, 0.49 (95% CI 0.28–0.85).

**Conclusions:**

In Asian and African patients with BCLC stage B/C hepatocellular carcinoma, HAIC—particularly the FOLFOX regimen—confers superior overall survival and oncologic outcomes compared to sorafenib, with higher response and disease control rates and reduced disease progression.

## 1. Introduction

Liver cancer, predominantly hepatocellular carcinoma(HCC) accounting for 75–85% of cases, ranked as the world’s sixth most diagnosed cancer and fourth top cancer death cause in 2018, with 841,000 new cases and 782,000 deaths [1–4]. There are many staging criteria for liver cancer, such as clinical staging, TNM staging, and Barcelona staging, Barcelona clinical liver cancer (BCLC) staging is a very commonly used method in clinical practice. Advanced BCLC stage B/C hepatocellular carcinoma, featuring larger tumors, vascular invasion, and metastases, is typically incurable with significantly shorter patient survival compared to early-stage BCLC A [5]. Sorafenib is a drug that inhibits multiple kinases, including those in the RAF/MEK/ERK signaling pathway.[6,7] Its efficacy in improving overall survival (OS) and prolonging time to disease progression has been proven in two Phase III randomized controlled trials for patients with advanced HCC [8,9]. Moreover, Sorafenib is still recommended as first-line systemic therapy for HCC with advanced liver cancer [10]. It can be seen that sorafenib is one of the important drugs in the treatment of advanced hepatocellular carcinoma. However, due to its poor solubility, rapid metabolism, and low bioavailability, the clinical application of sorafenib has been significantly limited [11].

In addition to sorafenib, Hepatic Artery Infusion Chemotherapy (HAIC) represents another treatment option for advanced-stage liver cancer [12]. HAIC employs catheter techniques to administer chemotherapeutic agents directly and continuously to the tumor site. As per earlier reports, HAIC stands out from systemic chemotherapy due to its heightened local control efficacy, accompanied by reduced systemic toxicity [13]. Recently, there has been a growing body of evidence supporting the efficacy of HAIC in treating advanced hepatocellular carcinoma [14–16], and HAIC has been adopted as an alternative therapy to sorafenib in Japan and several other Asian nations for the treatment of advanced hepatocellular carcinoma [17–19]. The benefits of HAIC as an alternative option for advanced hepatocellular carcinoma patients remain controversial. A previous systematic review and meta-analyse has compared the efficacy of sorafenib and HAIC in treating advanced hepatocellular carcinoma [20], whereas a direct comparison of their efficacy in BCLC stage B/C hepatocellular carcinoma has not yet been undertaken.

Consequently, the main objective of this systematic review and meta-analysis is to compare the efficacy and prognosis of HAIC versus sorafenib in BCLC stage B/C hepatocellular carcinoma patients, focusing on overall survival, tumor response, and adverse events, thereby providing practical guidance for clinical practice.

## 2. Materials and methods

### 2.1. Study protocol

This systematic review and meta-analysis were carried out following the Preferred Reporting Items for Systematic reviews and Meta-Analyses (PRISMA) [21]. This study did not require formal Institutional Review Board approval or patient informed consent, as it was a secondary analysis involving publicly available datasets. This meta-analysis was registered on PROSPERO (https://www.crd.york.ac.uk/PROSPERO/) with the registration number CRD42024538180.

### 2.2. Literature search

Literature searches were conducted in the EMBASE, PubMed, and Web of Science for studies published up until May 2024. Additional studies were identified through manual checks of references from included publications. The search strategy is detailed in S1 File.

### 2.3. Exclusion criteria and data extraction

The systematic review process was initiated by two independent reviewers, CZQ and LUT, who conducted the initial search, eliminated duplicate entries, and assessed titles and abstracts for relevance. They decided on the inclusion or exclusion of records based on this assessment. For the conclusive phase of screening, full texts were obtained for articles whose relevance couldn’t be definitively determined from their abstracts alone. Any disagreements regarding article selection were resolved through dialogue and consensus between the two reviewers. In instances where consensus was unattainable, a third reviewer, FYT, acted as an arbitrator to settle the discrepancies, ensuring a consistent and fair selection process.

The following criteria were used to include studies in the meta-analysis:(i) Patients confirmed to have HCC in BCLC B/C in the study population; (ii) Assessing the effectiveness of HAIC versus sorafenib; (iii) The study population consisting solely of humans; (iv) Studies that can provide data on Overall survival, Progression-Free-Survival (PFS), tumor response rates, and adverse reaction incidence. No limitations were imposed on the sample size or the duration of follow-up. when BCLC staging is not explicitly reported but can be reasonably inferred from clinical features (e.g., presence of portal vein tumor thrombosis or macrovascular invasion), a clinically pragmatic classification will be applied.

Tumor responses (including Complete Response (CR), Partial Response (PR), Stable Disease (SD), Progressive Disease (PD), Overall Response Rate (ORR), and Disease Control Rate (DCR)) and Progression-Free-Survival were evaluated using the modified Response Evaluation Criteria in Solid Tumors (mRECIST) [22]. Overall Survival is the length of time from either the date of diagnosis or the start of treatment for a disease, such as cancer, that patients diagnosed with the disease are still alive. It is often used as a measure of the effectiveness of a particular treatment. Progression-Free Survival is defined as the length of time during and after the treatment of a disease, such as cancer, that a patient lives with the disease but it does not get worse. It is another important endpoint in clinical trials, indicating how long patients remain free from disease progression. Complete Response refers to the disappearance of all target lesions.. Partial Response is defined as at least a 30% decrease in the sum of diameters of target lesions, taking as reference the baseline sum diameters. Stable Disease means that the tumor neither grows nor shrinks significantly. Progressive Disease is defined as at least a 20% increase in the sum of diameters of target lesions, taking as reference the smallest sum on study. Overall Response Rate was defined as the sum of Complete Response and Partial Response. Meanwhile, Disease Control Rate was defined as the cumulative incidence of Complete Response, Partial Response, and Stable Disease. An Adverse Event (AE) refers to any undesirable experience associated with the use of a medical product, such as a drug or medical device, in patients. Adverse events (AEs) can range from mild symptoms to severe reactions and may occur during clinical trials or post-marketing surveillance.Adverse Events were categorized according to the Common Terminology Criteria for Adverse Events (CTCAE) established by the National Cancer Institute [23–25].

The exclusion criteria of this meta-analysis were as follows: (i) Patients undergoing therapies for HCC that differ from both Sorafenib and HAIC; (ii) Patients receiving combined therapy of sorafenib and HAIC; (iii) Lacking a comparator group; (iv) Editorials, letters, abstracts, reviews, and case reports. (v) Missing or Unextractable Outcome Data.

### 2.4. Data extraction

Two investigators, named CZQ and LYT, separately gathered information from selected full-text articles using a standardized data extraction form. Any discrepancies in the data collected were resolved through mutual discussion between the reviewers. From each study included, the following details were recorded: first author’s last name, year of publication, country, study design, sample size, number of patients, demographic data (gender and age), previous treatment, etiology, BCLC staging, Child-Pugh class, Eastern Cooperative Oncology Group (ECOG) performance status, alpha-fetoprotein (AFP) levels, transaminase levels, bilirubin levels, Des-Gamma-Carboxy Prothrombin levels, platelet count, macroscopic vascular invasion (MVI) status, extrahepatic spread, lesion size, number of focal lesions, and clinical outcomes(tumor response, adverse events, Overall survival and Progression-free survival).

### 2.5. Study quality assessment

The quality and risk of bias in the randomized controlled trials (RCTs) were assessed using Cochrane Bias Risk Assessment Tool. The quality of observational studies was evaluated based on the modified Newcastle-Ottawa Scale (NOS), which encompasses three criteria: participant selection, comparability of study groups, and assessment of outcomes. Studies were scored on a scale of 0–9 to categorize their quality. Studies receiving a score of 6 or higher were deemed to be of high quality. The results of the quality assessment are shown in S2 File.

### 2.6. Statistical analysis

The statistical analyses were carried out using R version 4.3.2 software. For analyzing tumor response and Adverse events, pooled odds ratios (ORs) along with 95% confidence intervals (CIs) were computed. Meanwhile, for the evaluation of Overall survival and Progression-free survival, pooled hazard ratios (HRs) accompanied by 95% CIs were determined.This meta-analysis primarily focuses on the combined analysis of outcome measures. If the relevant outcome data are missing, the studies will not be included in the analysis.

### 2.7. Heterogeneity exploration

The statistical heterogeneity among the combined estimates was evaluated using I² statistics, with an I² value of 25% indicating low heterogeneity, 50% suggesting moderate heterogeneity, and 75% signifying high heterogeneity. All analyses were conducted using a random effects model, without depending on heterogeneity levels, because research indicates it provides more stable outcome estimates compared to fixed effect models [26].

### 2.8. Publication bias

Publication bias was evaluated qualitatively by funnel plot and quantitatively by Begg ‘s and Egger’ s tests (S3 File). P < 0.05 was considered statistically significant.

### 2.9. Sensitivity analysis

In the sensitivity analysis, we sequentially omitted one study at a time to evaluate the influence of each individual study on the aggregate outcome. The outcomes characterized by minimal heterogeneity between studies were deemed as the robust findings of our study. The results of the sensitivity analysis are shown in S4 File.

## 3. Results

### 3.1. Search results and study selection

**Fig 1:**
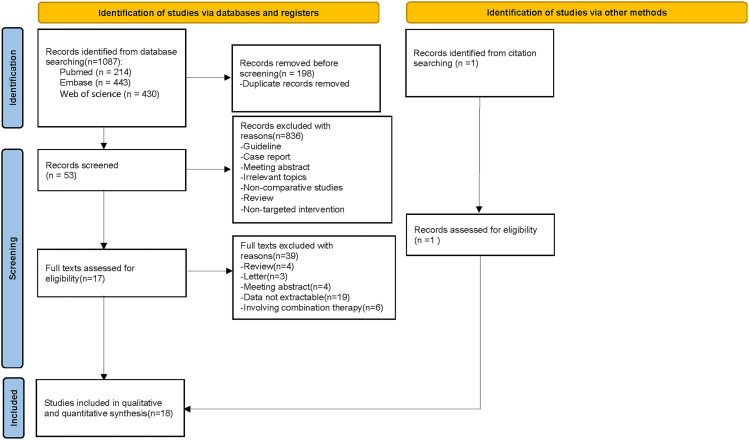
PRISMA flowchart.

Through database searching, 1087 relevant studies were identified, out of which 198 were found to be duplicates. After screening titles and abstracts, a total of 836 references were excluded. Following this, an additional 37 articles were eliminated during the full-text review stage. Upon scrutinizing the reference sections of all obtained articles, an extra study [27] was discovered that met the predetermined inclusion criteria. Finally, 18 studies [17,27–43] satisfied the inclusion and exclusion criteria and were deemed suitable for both qualitative and quantitative analyses, leading to their inclusion in the meta-analysis. The entire screening process is shown in Fig 1.

### 3.2. Study characteristics

The 18 selected studies were published within the most recent eight-year period, spanning from 2012 to 2022. Among the included studies, one was conducted in Egypt [35], five in Korea [17,27,39,41,43], eight in Japan [29,30,33,34,36,37,40,42], and four in China [28,31,32,38]. The 18 studies collectively encompassed 3008 patients, with 1520 patients (49.0%) having undergone HAIC, while 1568 patients (51.0%) had been administered sorafenib. According to the Newcastle-Ottawa Scale, the quality of the included literature ranged from 7 to 9 points. The quality of the studies incorporated was generally of a high quality. The major characteristics of the studies and the results of their quality assessment are presented in Table 1.

**Table 1:** Baseline characteristics.

Study (year)	Country	study type	HAIC’s agent	Treatment	Patients	Previous treatment (Absent/present)	Gender (M/F)	Mean age	Etiology HBV/HCV/others	Child Pugh (A/B/C)	ECOG PS (0/1/ > =2)	BCLC staging (B/C)	AFP (ng/ml)
Abdelmaksoud 2021	Egypt	CCS	Ad/C	HAIC sorafenib	20 29	NA NA	18/2 26/3	NA NA	NA NA	20/0/0 20/0/0	NA NA	0/20 0/29	1,942.53 ± 5,715.44 26,102.58 ± 8,0097.45
Yang 2017	Korea	R	C/5-FU	HAIC sorafenib	54 53	27/27 16/37	50/4 39/14	54.4 ± 11.0 58.0 ± 9.2	44/6/4 43/4/6	25/29/0 34/19/0	NA NA	0/54 0/53	23/31(<400/ ≥ 400ng/dl) 23/30 (<400/ ≥ 400ng/dl)
Hatooka 2016	Japan	R	C/5-FU or 5-FU/IFN	HAIC sorafenib	48 48	0/48 0/48	42/6 36/12	68 (46-83) 68 (50-88)	11/32/5 12/33/3	48/0/0 48/0/0	46/2/0 45/3/0	42/6 42/6	415.3 (5-191500) 305 (5-12230)
Huang 2022	China	R	FOLFOX	HAIC sorafenib	55 59	0/55 0/59	50/5 56/3	NA NA	NA NA	55/0/0 59/0/0	16/39/0 24/35/0	35/20 41/18	21/34 (≤1000/> 1000ng/ml) 23/36 (≤1000/> 1000ng/ml)
Iwamoto 2021	Japan	R	New-FP	HAIC sorafenib	344 344	NA NA	267/77 271/73	68.50 ± 11.02 68.35 ± 10.33	NA NA	244/98/2 252/90/2	NA NA	70/274 89/255	598.04 ± 358.41 619.26 ± 342.57
Iwamoto 2022	Japan	R	New-FP	HAIC sorafenib	198 198	NA NA	NA NA	66.6 ± 10.9 65.3 ± 10.7	NA NA	198/0/0 198/0/0	NA NA	46/152 50/148	48,645.45 ± 248,475.48 52,073.81 ± 163,296.82
Jeong 2012	Korea	R	C/5-FU	HAIC sorafenib	21 20	NA NA	21/0 11/9	51.0 (33–75) 59.5 (49–75)	NA NA	10/11/0 14/6/0	0/19/2 0/16/4	0/21 0/20	12/9 (≥400/ ＜ 400ng/ml) 13/7 (≥400/<400ng/ml)
Kawaoka 2015	Japan	R	C/5-FU or 5-FU/IFN	HAIC sorafenib	16 16	2/14 2/14	16/0 13/3	65 (40–85) 64 (30–81)	2/11/3 8/6/2	16/0/0 16/0/0	NA NA	0/16 0/16	33 (5–165 500) 208 (3–85 632)
Kondo 2015	Japan	R	C	HAIC sorafenib	44 83	0/44 0/83	32/12 74/9	71 (54–84) 70 (37–88)	1/33/10 16/52/15	31/13/0 78/5/0	NA NA	16/28 58/25	588.5 (3–207890) 199.7 (1.6–529490)
Li 2020	China	R	FOLFOX	HAIC sorafenib	47 47	45/2 41/6	41/6 45/2	52 (25–78) 51.6 ± 2.5	NA NA	44/3/0 44/3/0	NA NA	0/47 0/47	26/21 (≤400/> 400ng/ml) 18/29 (≤400/> 400ng/ml)
Lyu 2021	China	RCT	FOLFOX	HAIC sorafenib	130 132	113/17 113/19	115/15 123/9	54 (45-61) 53 (45-62)	120/2/8 144/4/14	88/42/0 93/39/0	15/83/32 14/95/23	5/125 9/123	337.8 (28.5−12,902.75) 304.2 (15.3−3,086.5)
Lyu 2018	China	R	FOLFOX	HAIC sorafenib	147 147	101/46 98/49	133/14 133/14	52 (25–77) 50 (16–82)	128/13/6 130/9/8	94/53/0 98/49/0	10/72/65 7/77/63	4/143 2/145	478.4 (1.8–121000) 478.4 (1.8–121000)
Song 2015	Korea	R	C/5-FU or C/5-FU/EPI	HAIC sorafenib	50 60	31/19 39/21	38/12 44/16	54.3 ± 9.9 55.8 ± 9.0	44/2/4 41/5/14	5/45/0 13/47/0	NA NA	0/50 0/60	15/35 (<200/ ≥ 200ng/dl) 20/38 (<200/ ≥ 200ng/dl)
Zaizen 2021	Japan	R	C	HAIC sorafenib	83 83	NA NA	56/27 60/23	73.7 ± 9.6 73.2 ± 10.0	6/67/10 7/65/11	53/30/0 59/24/0	NA NA	71/12 74/9	75 (2-222500) 100 (3-177630)
Shiozawa 2015	Japan	R	C/5-FU	HAIC sorafenib	26 17	0/26 0/17	22/4 14/3	66.2 ± 6.3 69.5 ± 6.6	5/20/1 2/10/5	20/6/0 13/4/0	NA NA	21/5 14/3	10341.2 2690.2
Ahn 2021	Korea	R	C/5-FU	HAIC sorafenib	38 35	29/9 21/14	30/8 30/5	53.0 ± 11.6 58.3 ± 9.5	33/2/3 24/2/9	27/11/0 24/11/0	29/7/2(0-1/2/3) 24/9/2(0-1/2/3)	0/38 0/35	71,341 ± 14,823 69,745 ± 21,274
Ueshima 2020	Japan	R	C/5-FU or 5-FU/IFN or C	HAIC sorafenib	170 170	77/93 88/82	141/29 130/40	67.3 68.9	90/35/45 75/40/55	133/37(A/ ≥ B) 133/37(A/ ≥ B)	NA NA	0/170 0/170	72/98 (≤400/ > 400ng/ml) 72/98 (≤400/> 400ng/ml)
Choi 2018	Korea	RCT	C/5-FU	HAIC sorafenib	29 29	20/9 26/3	25/4 27/2	60.3 ± 9.5 60.2 ± 7.3	21/0/8 18/5/6	27/2 25/4	NA NA	0/29 0/29	260.0 (3.6–84604.6) 130.8 (2.0–225971)
**Study (year)**	**DCP** **(mAU/ml)**	**ALT, U/L**	**AST, U/L**	**Total bilirubin** **,mg/L**	**Platelet count, × 109/L**		**Macroscopic vascular invasion** **(Absent/present)**		**Extrahepatic spread** **(Absent/present)**	**Lesion size** **(cm)**	**No. of focal lesions** **(Solitary/Multiple)**		**Quality**
Abdelmaksoud 2021	NA NA	NA NA	NA NA	0.93 ± 0.52 1.82 ± 2.28	134.93 ± 46.29 166.55 ± 67.38		NA NA		NA NA	5.19 ± 2.66 5.83 ± 2.05	13/7 14/15		6
Yang 2017	NA NA	NA NA	NA NA	NA NA	NA NA		0/54 0/53		26/28 18/35	12.5 ± 4.6 9.2 ± 5.1	8/46 17/36		7
Hatooka 2016	370 (7-16163) 414 (14-58970)	NA NA	NA NA	NA NA	122 (46-888) 153 (53-207)		0/48 0/48		48/0 48/0	3.8 (1.5-14.0) 3.3 (1.0-8.0)	9/39 9/39		8
Huang 2022	NA NA	NA NA	NA NA	NA NA	NA NA		37/18 42/17		NA NA	23/32 (≤10/> 10 cm) 32/27 (≤10/>10 cm)	12/43 13/46		9
Iwamoto 2021	589.06 ± 342.95 612.01 ± 365.21	NA NA	NA NA	NA NA	NA NA		85/269 84/260		230/114 222/122	10.974 ± 5.342 10.749 ± 5.458	NA NA		8
Iwamoto 2022	30,379.13 ± 95,631.52 27,714.78 ± 61,755.77	NA NA	NA NA	NA NA	NA NA		55/143 52/146		NA NA	7.62 ± 4.21 7.26 ± 4.75	NA NA		8
Jeong 2012	NA NA	NA NA	NA NA	NA NA	NA NA		NA NA		NA NA	NA NA	NA NA		6
Kawaoka 2015	3292 (39–102 590) 4692 (14–348 650)	NA NA	NA NA	NA NA	122 (46–888) 153 (53–207)		0/16 0/16		NA NA	5.0 (1.5–12.0) 5.0 (1.0–19.0)	NA NA		8
Kondo 2015	461 (9–75000) 340 (14–893000)	48.5 (16–332) 47 (12–213)	70.5 (20–339) 62 (19–341)	0.9 (0.2–2.0) 0.8 (0.3–2.6)	NA NA		19/25 61/22		0/44 0/83	NA NA	NA NA		7
Li 2020	NA NA	44.8 (10.2–170.8) 51 (13.8–220.6)	NA NA	3.040 (3.040–19.763) 4.210 (4.210–22.374)	237 (72–664) 152 (10.7–512)		5/42 3/44		35/12 35/12	9.8 ± 0.5 7.7 ± 0.8	9/38 9/38		6
Lyu 2021	NA NA	NA NA	NA NA	NA NA	NA NA		36/94 41/91		86/44 86/46	11.7 (8.3-14.0) 10.8 (8.7-13.6)	NA NA		*
Lyu 2018	NA NA	NA NA	NA NA	NA NA	NA NA		65/82 67/80		72/75 67/80	11.7 ± 3.9 11.7 ± 3.8	NA NA		9
Song 2015	NA NA	NA NA	NA NA	NA NA	NA NA		0/50 0/60		38/12 39/21	22/28 (<10/ ≥ 10 cm) 31/29 (<10/ ≥ 10 cm)	NA NA		8
Zaizen 2021	316 (6–344,000) 582 (11–112,000)	NA NA	NA NA	9 (3–27) 10 (3–29)	NA NA		71/12 74/9		NA NA	NA NA	NA NA		8
Shiozawa 2015	14066.8 2737.2	NA NA	NA NA	NA NA	NA NA		NA NA		NA NA	NA NA	NA NA		8
Ahn 2021	1,361 ± 821 1,437 ± 824	58 ± 35 56 ± 47	89 ± 63 103 ± 98	1.04 ± 0.48 1.16 ± 0.55	162 ± 83 196 ± 105		0/38 0/35		25/13 21/14	NA NA	NA NA		8
Ueshima 2020	NA NA	NA NA	NA NA	NA NA	NA NA		106/64 106/64		NA NA	123/47(≤50/ > 50 mm) 123/47(≤50/ > 50 mm)	66/104(≤3/ > 3) 66/104(≤3/ > 3)		8
Choi 2018	NA NA	NA NA	NA NA	NA NA	NA NA		0/29 0/29		NA NA	14/15 (<10/ ≥ 10 cm) 12/17 (<10/ ≥ 10 cm)	13/16 10/19		*

F female, M male, AFP alpha fetoprotein, ECOG PS Eastern Cooperative Oncology Group Performance Status Scale, NA not available, EPI epirubicin, Ad adriamycin, C cisplatin, 5-FU 5-fluorouracil, FOLFOX oxaliplatin/5-fluorouracilleucovorin, New-FP the fine-powderCDDP (DDP-H, IA-Call, Nippon Kayaku, Tokyo, Japan)/ethiodized oil/ 5-FU, DCP Des-γ-Carboxy Prothrombin, AST Aspartate Aminotransferase, ALT Alanine Aminotransferase.

### 3.3. Results of the meta-analysis

#### 3.3.1. Overall survival and progression-free survival.

**Fig 2:**
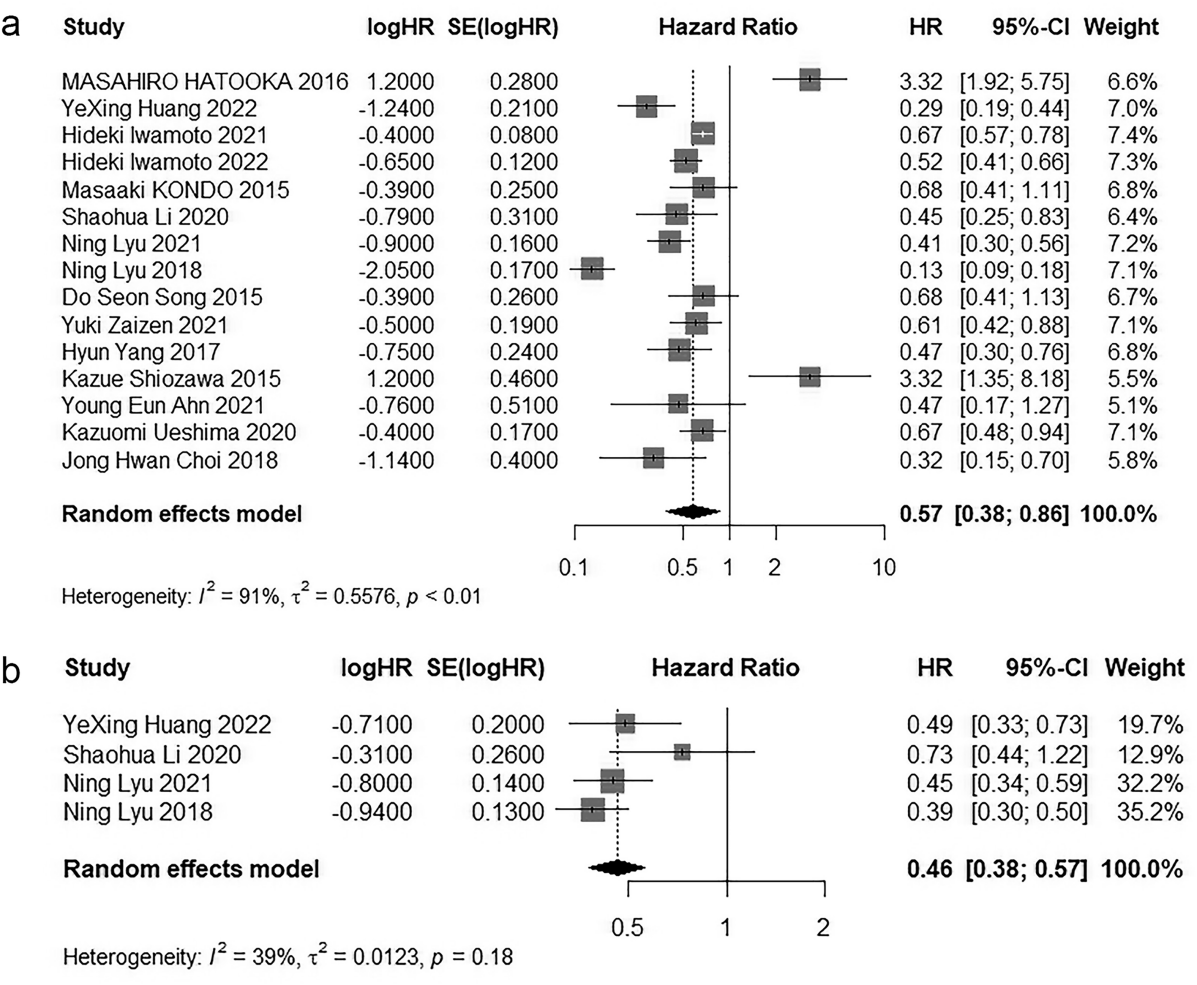
Forest plot for the comparison of OS and PFS. **a** Overall survival, **b** Progression-free survival.

Among the 18 included studies, 15 studies [27–34,36,38–43] reported Hazard Ratio for Overall survival. Through a meta-analysis employing a random-effects model, the combined Hazard Ratio was calculated to be 0.57 (95% CI = 0.38–0.86, P < 0.05). In addition, 4 studies [28,31,32,38] included studies reported Hazard Ratio for Progression-free survival. Through a random-effects model, the combined Hazard Ratio was calculated to be 0.46 (95% CI = 0.38–0.57, P < 0.05). The forest plots for Progression-free survival and Overall survival are depicted in Fig 2.

#### 3.3.2. Subgroup analysis for OS by treatment regimen.

**Fig 3:**
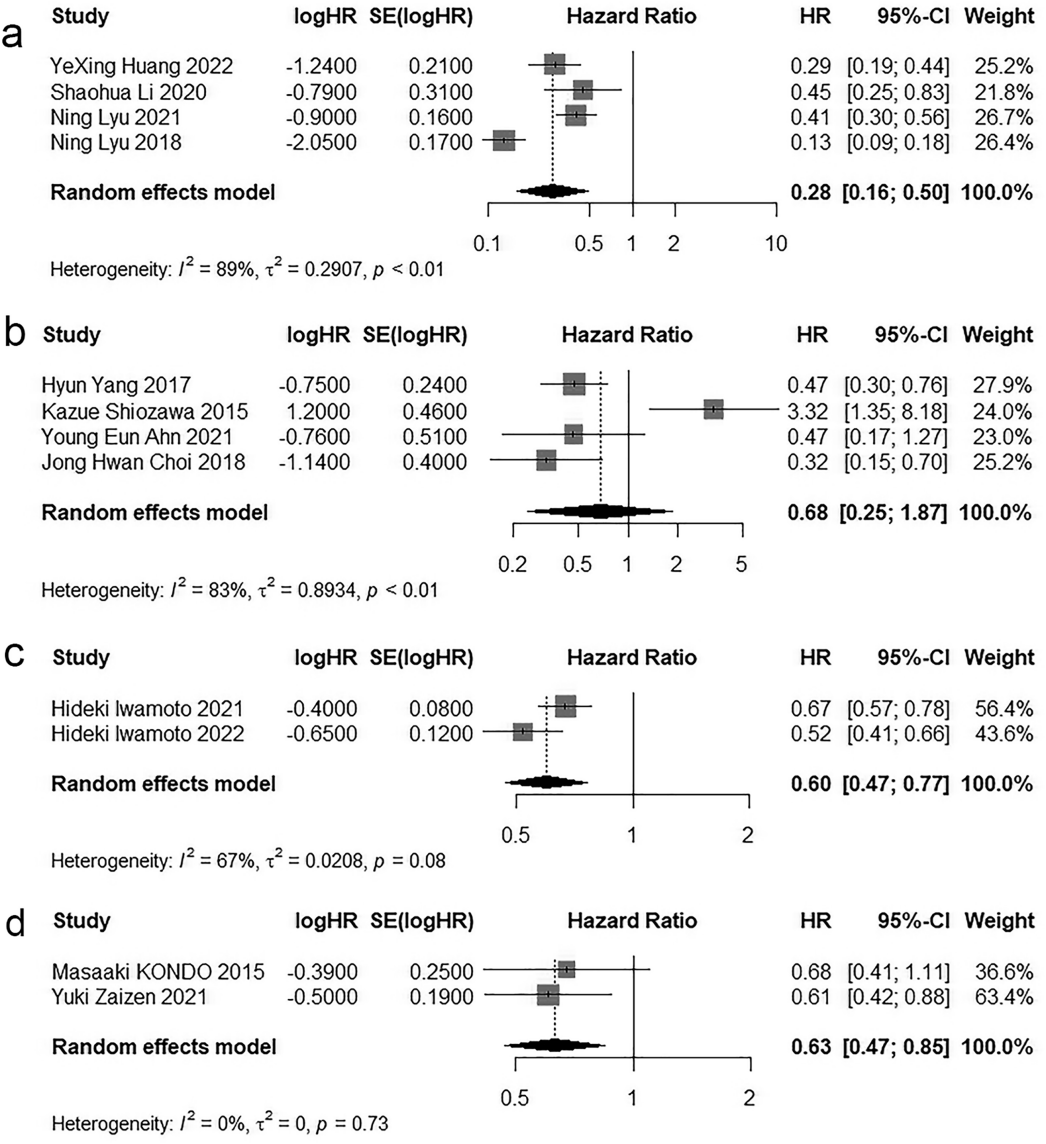
Forest plot for subgroup analyses by treatment regimen. **a** FOLFOX-based HAIC regimens, **b** FP regimen, **c** New-FP regimen, **d** cisplatin-based HAIC.

Among the 18 included studies, four studies exclusively enrolled patients receiving FOLFOX-based HAIC regimens. A meta-analysis using a random-effects model yielded a pooled hazard ratio of 0.28 (95% CI: 0.16–0.50; *P* < 0.05). Four studies exclusively used cisplatin plus 5-FU-based HAIC(FP regimen), with a pooled HR of 0.68 (95% CI: 0.25–1.87; *P* > 0.05) from the random-effects meta-analysis. Two studies exclusively employed fine-powder cisplatin-based HAIC (New-FP regimen), resulting in a pooled HR of 0.60 (95% CI: 0.47–0.77; *P* < 0.05). Additionally, two studies exclusively utilized cisplatin-based HAIC, showing a pooled HR of 0.63 (95% CI: 0.47–0.85; *P* < 0.05). Forest plots for these subgroup analyses by treatment regimen are presented in Fig 3.

#### 3.3.3. Tumor response rate.

**Fig 4:**
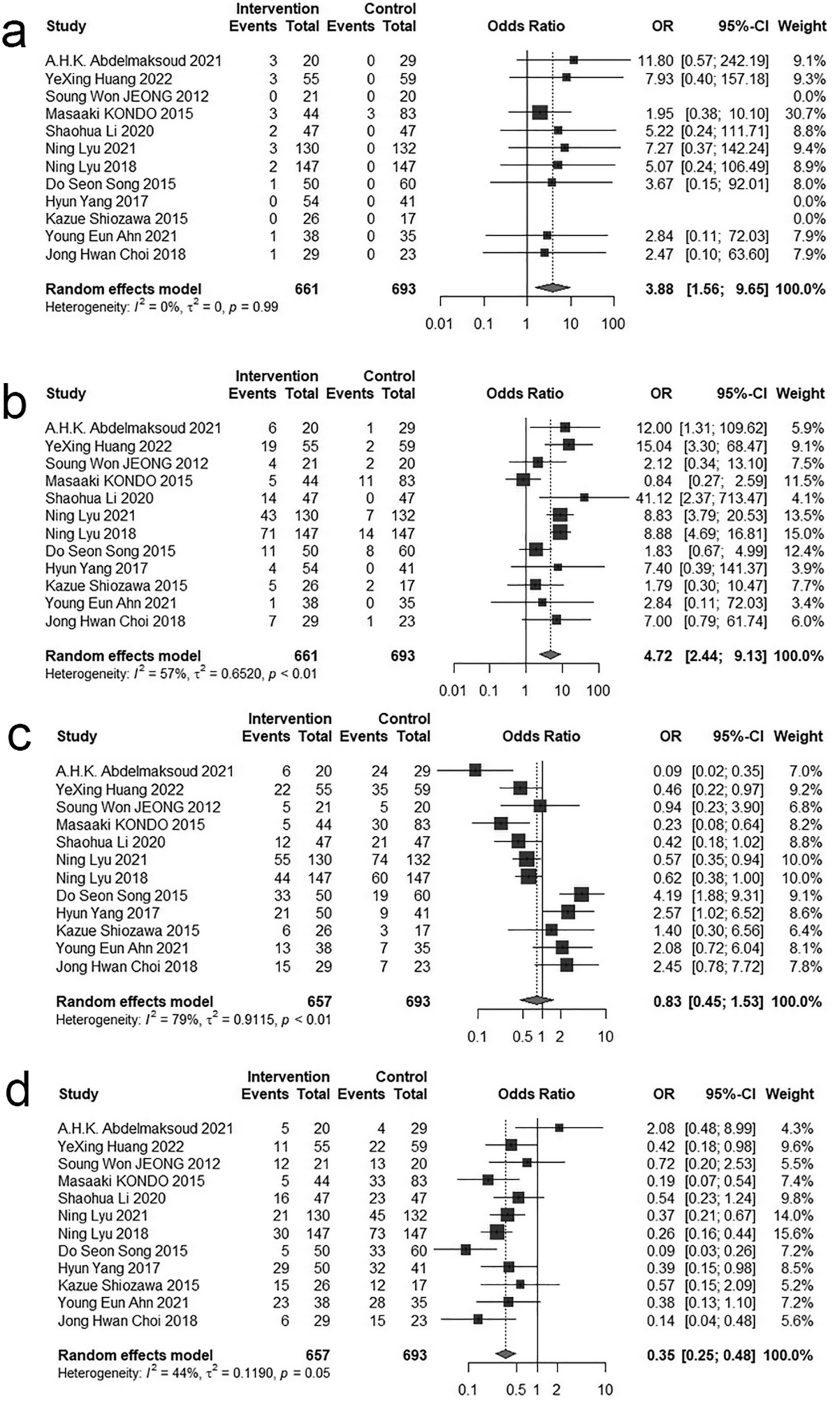
Forest plot for the comparison of CR, PR, SD and PD. **a** Complete response, **b** Partial response, **c** Stable disease, **d** Progressive disease.

**Fig 5:**
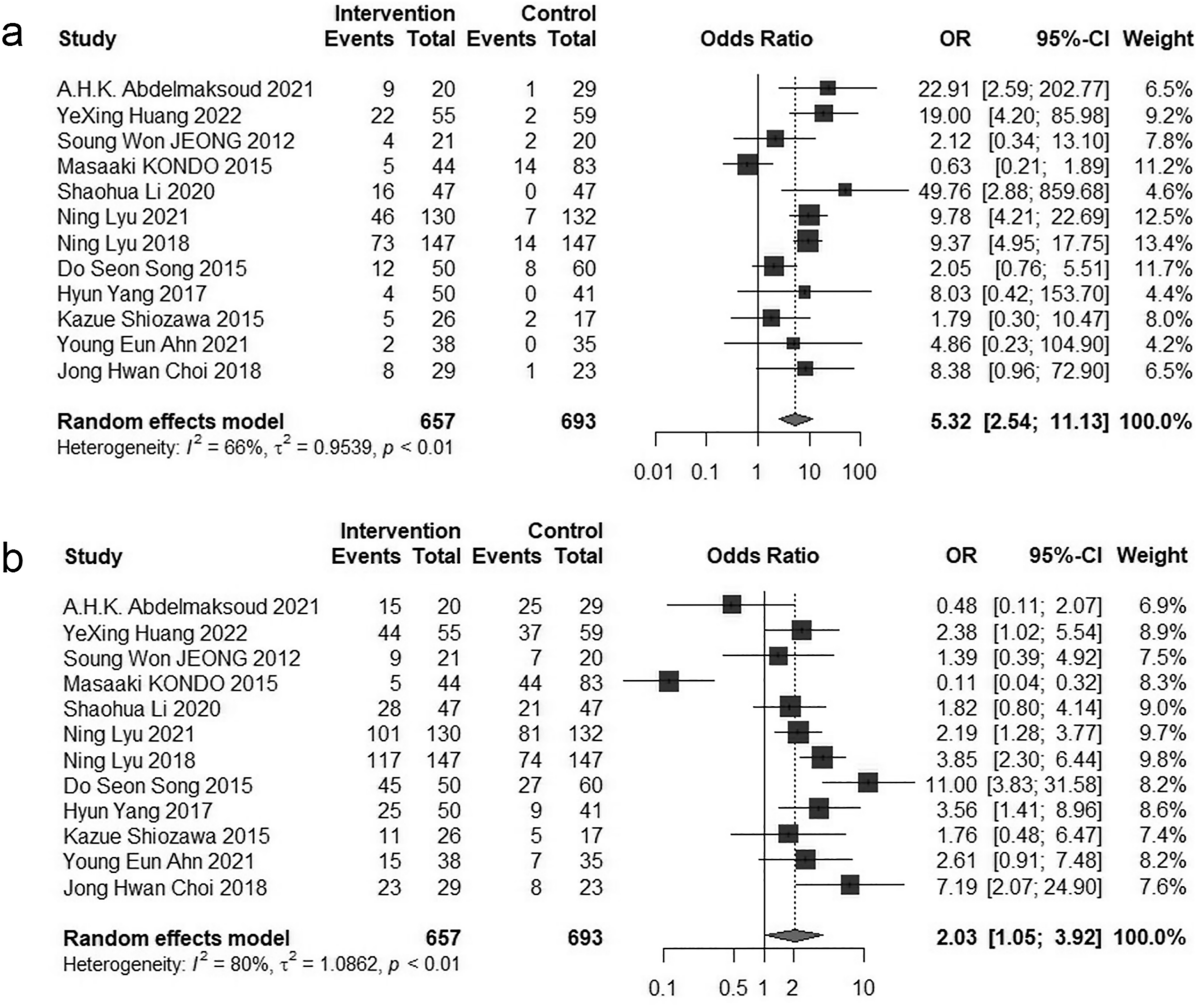
Forest plot for the comparison of ORR and DCR. **a** Objective response rate, **b** Disease control rate.

Twelve studies [17,27,28,31,32,34,35,38–41,43] compared the tumor response rates between HAIC and sorafenib. A random-effects model was employed to calculate the pooled effect sizes. The combined Odds Ratio for Complete response was 3.88 (95% CI = 1.56–9.65, P < 0.05). For Partial response, the combined Odds Ratio was 4.72(95% CI = 2.44–9.13, P < 0.05). Stable disease showed a combined Odds Ratio of 0.83 (95% CI = 0.45–1.53, P > 0.05). Progressive disease had a combined Odds Ratio of 0.35 (95% CI = 0.25–0.48, P < 0.05). The combined Odds Ratio for Objective response rate was 5.32 (95% CI = 2.54–11.13, P < 0.05), while the Disease control rate demonstrated a combined Odds Ratio of 2.03 (95% CI = 1.05–3.92, P < 0.05). The Forest plots for tumor response rate are showed in Fig 4 and Fig 5.

#### 3.3.4. Adverse events.

**Fig 6:**
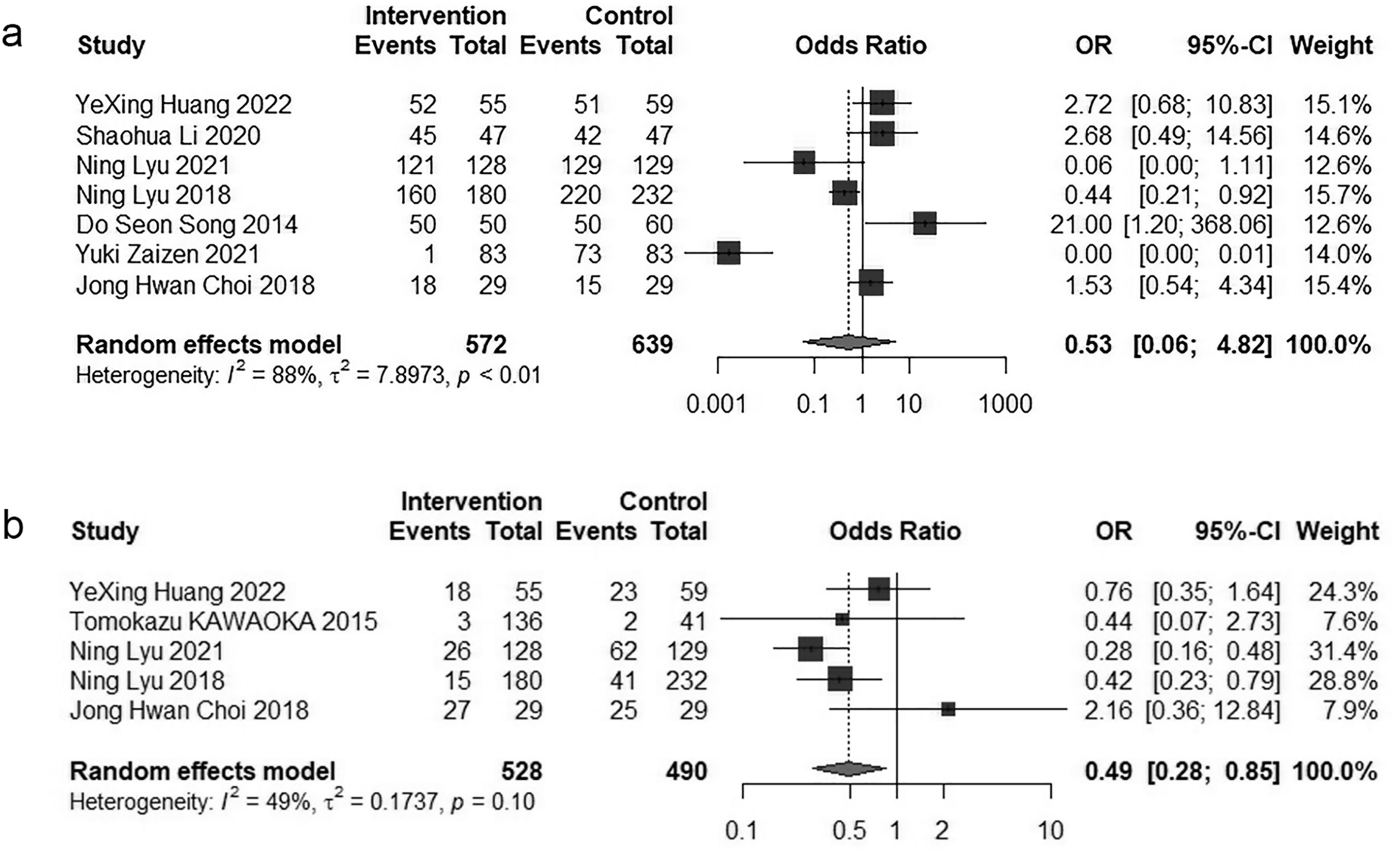
Forest plot for the comparison of AEs and 3-4 level AEs. **a** Adverse events, **b** 3-4 level Adverse events.

This meta-analysis compared the incidence rates of adverse events, including both overall occurrences and those graded as 3−4, between two therapeutic approaches, and summarized the reported adverse events. Among the studies included, seven studies [28,31–33,38,39] provided data on the total number of patients who experienced any adverse event, while five studies [31,32,37,38,43] specifically reported figures for patients encountering grade 3−4 adverse events. Utilizing a random-effects model, the combined Odds Ratio for the overall incidence of adverse events was estimated at 0.53 (95% CI = 0.06–4.82, P > 0.05). Conversely, the pooled Odds Ratio for the incidence of grade 3−4 adverse events was 0.49 (95% CI = 0.28–0.85, P < 0.05). The Forest plots are showed in Fig 6. In Table 2, we provide a summary of detailed studies on treatment-related adverse events, including complications involving the hematologic system, dermatologic system, digestive system, hepatic and renal function, as well as systemic symptoms. Adverse events associated with HAIC are predominantly hematologic in nature; five studies [27,31,34,39,41] reported higher incidences of neutropenia, anemia, and thrombocytopenia in the HAIC group compared to the Sorafenib group. In contrast, Sorafenib-related adverse events are primarily dermatologic, with five studies [27,31,32,38,39] demonstrating a higher incidence of hand-foot syndrome, alopecia, and rash in the Sorafenib group relative to the HAIC group.

**Table 2:** Treatment-related adverse reactions.

Adverse events	Yang 2017				Huang 2022				Kondo 2015				Lyu 2021				Lyu 2018				Song 2015				Ahn 2021				Choi 2018			
	HAIC(n = 54)		Sorafenib (n = 53)		HAIC (n = 55)		Sorafenib (n = 59)		HAIC (n = 44)		Sorafenib (n = 83)		HAIC (n = 129)		Sorafenib (n = 128)		HAIC (n = 180)		Sorafenib (n = 232)		HAIC (n = 50)		Sorafenib (n = 60)		HAIC (n = 38)		Sorafenib (n = 35)		HAIC (n = 29)		Sorafenib (n = 29)	
	No.	%	No.	%	No.	%	No.	%	No.	%	No.	%	No.	%	No.	%	No.	%	No.	%	No.	%	No.	%	No.	%	No.	%	No.	%	No.	%
**Hematological**																																
Neutropenia	26	0.48	0	0.00	15	0.27	9	0.15	3	0.07	1	0.01	33	0.26	47	0.37	39	0.22	89	0.38	34	0.68	0	0	9	0.24	1	0.03	3	0.10	NA	NA
Anemia	3	0.06	0	0.00	22	0.40	18	0.31	1	0.02	1	0.01	41	0.32	41	0.32	14	0.08	129	0.56	49	0.98	0	0	14	0.37	7	0.20	2	0.07	NA	NA
Thrombocytopenia	12	0.22	0	0.00	20	0.36	8	0.14	5	0.11	8	0.10	45	0.35	35	0.27	33	0.18	117	0.50	42	0.84	0	0	17	0.45	3	0.09	1	0.03	NA	NA
**Dermatologic events**																																
Hand and foot syndrome	0	0.00	15	0.28	1	0.02	27	0.46	0	0.00	13	0.16	0	0.00	68	0.53	0	0.00	71	0.31	0	0.00	27	0.45	0	0.00	10	0.29	NA	NA	9	0.31
Alopecia	0	0.00	5	0.09	2	0.04	14	0.24	NA	NA	NA	NA	2	0.02	19	0.15	0	0.00	33	0.14	0	0.00	42	0.70	NA	NA	NA	NA	1	0.03	3	0.10
Rash	NA	NA	NA	NA	2	0.04	16	0.27	1	0.02	1	0.01	1	0.01	26	0.20	0	0.00	37	0.16	0	0.00	14	0.23	0	0.00	1	0.03	NA	NA	NA	NA
**Gastrointestinal**																																
Loss of appetite	NA	NA	NA	NA	NA	NA	NA	NA	0	0.00	10	0.12	12	0.09	22	0.17	10	0.06	47	0.20	NA	NA	NA	NA	5	0.13	9	0.26	NA	NA	1	0.03
Abdominal pain	NA	NA	NA	NA	22	0.40	2	0.03	NA	NA	NA	NA	52	0.40	18	0.14	79	0.44	68	0.29	NA	NA	NA	NA	2	0.05	3	0.09	NA	NA	NA	NA
Nausea	NA	NA	NA	NA	34	0.62	14	0.24	1	0.02	0	0.00	24	0.19	28	0.22	27	0.15	58	0.25	NA	NA	NA	NA	4	0.11	7	0.20	NA	NA	NA	NA
Diarrhea	NA	NA	NA	NA	20	0.36	24	0.41	2	0.05	3	0.04	27	0.21	47	0.37	24	0.13	129	0.56	0	0.00	23	0.38	2	0.05	6	0.17	2	0.07	5	0.17
Vomiting	NA	NA	NA	NA	32	0.58	12	0.20	1	0.02	0	0.00	29	0.22	21	0.16	14	0.08	41	0.18	NA	NA	NA	NA	4	0.11	7	0.20	NA	NA	NA	NA
**Liver dysfunction**																																
Elevated ALT	9	0.17	9	0.17	33	0.60	40	0.68	7	0.16	7	0.08	28	0.22	26	0.20	54	0.30	44	0.19	37	0.74	0	0.00	5	0.13	2	0.06	9	0.31	3	0.10
Elevated AST	NA	NA	NA	NA	38	0.69	43	0.73	7	0.16	13	0.16	58	0.45	35	0.27	103	0.57	71	0.31	NA	NA	NA	NA	6	0.16	8	0.23	10	0.34	8	0.28
Hyperbilirubinemia	16	0.30	19	0.36	31	0.56	25	0.42	2	0.05	6	0.07	22	0.17	46	0.36	19	0.11	117	0.50	37	0.74	0	0.00	6	0.16	13	0.37	13	0.45	10	0.34
Hypoalbuminemia	NA	NA	NA	NA	40	0.73	20	0.34	NA	NA	NA	NA	36	0.28	38	0.30	57	0.32	97	0.42	NA	NA	NA	NA	NA	NA	NA	NA	NA	NA	NA	NA
**Constitutional symptoms**																																
Fatigue	0	0.00	5	0.09	30	0.55	23	0.39	0	0.00	2	0.02	32	0.25	46	0.36	125	0.69	99	0.43	0	0.00	22	0.36	3	0.08	10	0.29	NA	NA	NA	NA
Weight loss	NA	NA	NA	NA	13	0.24	19	0.32	NA	NA	NA	NA	20	0.16	38	0.30	50	0.28	65	0.28	NA	NA	NA	NA	NA	NA	NA	NA	NA	NA	NA	NA
**Hypertension**	NA	NA	NA	NA	2	0.04	13	0.22	0	0.00	4	0.05	1	0.01	35	0.27	2	0.01	87	0.38	0	0.00	18	0.30	NA	NA	NA	NA	NA	NA	NA	NA
**Elevated creatinine**	4	0.07	1	0.02	NA	NA	NA	NA	0	0.00	0	0.00	3	0.02	32	0.25	6	0.03	66	0.28	NA	NA	NA	NA	NA	NA	NA	NA	1	0.03	NA	NA

### 3.4. Sensitivity analysis and publication bias

We conducted a sensitivity analysis on the pooled Overall survival values. The sensitivity analysis demonstrated that the outcomes for every endpoint remained consistent and unaffected by the removal of any single study, confirming their stability. The results of sensitivity analysis are depicted in S4 File. Publication bias analysis was confined solely to the pooled Overall survival values in this article, as the number of studies included in other evaluation groups was less than 10. In the meta-analyses of Overall survival, no substantial evidence of publication bias was detected, as inferred from the results of the formal statistical tests applied (for Overall survival: Egger’s test, P-value = 0.8828; Begg’s test, P-value = 0.7662). The funnel plot of the primary outcome displayed symmetry, implying a low likelihood or absence of publication bias (S3 File).

## 4. Discussion

This study constitutes a systematic review and meta-analysis, aimed at further evaluating the efficacy and safety of sorafenib versus HAIC in the treatment of BCLC B/C stage HCC. Our findings indicate that, for patients with BCLC B/C stage HCC, HAIC demonstrates more favorable outcomes compared to sorafenib in terms of Overall survival, Progression-free survival, Complete response, Partial response, Progressive disease, Objective response rate, and Adverse events. A previous meta-analysis [20] had already compared the efficacy and prognosis of sorafenib versus HAIC in advanced HCC. Given that the BCLC staging system is commonly employed in clinical practice and there remains controversy surrounding the treatment of BCLC B/C stage HCC, our meta-analysis specifically focuses on this patient subgroup. Additionally, this study also conducted subgroup analyses according to different HAIC regimens and evaluated tumor response, including complete response, partial response, stable disease, and progressive disease. Furthermore, the incidence of adverse events, as well as grade 3–4 adverse events, was compared to inform future clinical practice.

The most significant discovery in this meta-analysis’s comparison of therapeutic effects is that BCLC B/C stage patients receiving HAIC demonstrated superior Overall survival relative to those treated with sorafenib. In addition to this, the HAIC group had better Progression-free survival compared to the sorafenib group. In terms of tumor response rates, HAIC demonstrated significantly superior efficacy compared to sorafenib, with higher rates of complete response and partial response, a lower rate of disease progression, and significantly improved objective response rate and disease control rate. However, no statistically significant disparities were evident between HAIC and sorafenib in terms of Stable disease. The aforementioned findings are based on a random-effects model. Conversely, when adopting a fixed-effect model, the pooled results for Stable disease alters to 0.77 (95% CI = 0.62–0.96, P ＜ 0.05) (S5 File). This discrepancy underscores the probable impact of substantial heterogeneity among the studies, necessitating future meta-analyses to incorporate additional research to validate HAIC’s superiority in terms of Stable disease and Disease control rate. We contend that HAIC may confer certain advantages over sorafenib in terms of survival, tumor progression, and tumor response, grounded in several rationales. Firstly, the liver is endowed with dual blood supplies: the portal vein and the hepatic artery; HCC predominantly depends on the branches of the hepatic artery for its blood supply [44,45]. Unlike systemic drug administration, HAIC facilitates the direct, high-concentration delivery of chemotherapeutic agents to the tumor site, thereby enabling a heightened local concentration of anti-cancer drugs, reducing systemic dissemination, and thereby augmenting the potency of these medications. Secondly, given that liver function in HCC patients tends to be impaired, HAIC is tolerable for those with poorer liver function due to its lower toxicity profile, effectively managing tumor progression through both anti-angiogenic and anti-proliferative effects. Lastly, the genetic heterogeneity among HCC cells stemming from diverse etiologies significantly contributes to inherent resistance to sorafenib, which is a pivotal factor undermining the therapeutic effectiveness of sorafenib in some patients [46–48]. In recent years, a randomised phase 3 non-inferiority trial [10] has uncovered that lenvatinib exhibits remarkable efficacy in treating advanced hepatocellular carcinoma, suggesting it as a potential alternative to sorafenib as a systemic therapy option.

Furthermore, given the heterogeneity in HAIC regimens across the included studies—including FOLFOX, FP, New-FP, and cisplatin monotherapy—we performed a prespecified subgroup analysis by chemotherapy backbone. Compared with sorafenib, FOLFOX-based HAIC significantly improved OS (HR = 0.28; 95% CI: 0.16–0.50; *P* < 0.05). New-FP–based HAIC (HR = 0.60; 95% CI: 0.47–0.77; *P* < 0.05) and cisplatin-based HAIC (HR = 0.63; 95% CI: 0.47–0.85; *P* < 0.05) also showed significant OS benefits. The FP regimen demonstrated a favorable trend (HR = 0.68; 95% CI: 0.25–1.87), though not statistically significant, suggesting a need for further validation. These results highlight the importance of regimen selection in HAIC and support FOLFOX as the most evidence-backed HAIC approach for first-line treatment of advanced HCC versus sorafenib. The efficacy of FOLFOX in intermediate-to-advanced or unresectable HCC is largely attributable to its synergy with HAIC. As HCC— including tumors with portal vein tumor thrombus—is primarily hepatic artery–fed, HAIC delivers sustained, high local concentrations of oxaliplatin plus 5-FU/leucovorin, enhancing intrahepatic tumor control while minimizing systemic toxicity and enabling patients with compromised liver function to better tolerate full-course therapy [49]. This “intensified local control” is supported by high-level evidence: the phase III FOHAIC-1 trial showed that HAIC-FOLFOX significantly improved survival and tumor response versus sorafenib, particularly in patients with macrovascular invasion, underscoring its clinical value in advanced HCC driven by high intrahepatic burden and vascular involvement [50].

Sorafenib and HAIC are both first-line options for intermediate- to advanced-stage HCC. Sorafenib has been the standard systemic therapy since 2007 [51], while HAIC has been widely used in Japan since the early 2000s and remains strongly recommended in Japanese and Chinese guidelines for advanced HCC [52,53]. Our study shows that HAIC provides better outcomes than sorafenib, supporting its role as a valid first-line treatment. Recent evidence further suggests that combining HAIC with immunotherapy—typically FOLFOX-HAIC plus PD-1/PD-L1 inhibitors, often with a TKI (triplet regimen)—yields promising efficacy in unresectable or advanced HCC. A phase II trial of lenvatinib + toripalimab + HAIC reported high ORR, prolonged survival, and manageable toxicity [54]. Real-world multicenter studies also confirm that HAIC-based combo therapy significantly improves ORR, PFS, and OS in high-risk patients (e.g., with PVTT or infiltrative HCC) and increases resection conversion rates, with acceptable safety [55–58]. While more RCTs are needed to refine regimens, identify biomarkers, and assess long-term safety, HAIC combined with immunotherapy is poised to become a new standard for advanced HCC. In future work, we will continue to investigate the comparative efficacy of HAIC versus other currently recommended first-line strategies—such as HAIC versus TACE or HAIC versus lenvatinib—and further explore the therapeutic potential of HAIC integrated into multimodal “targeted-immunotherapy” regimens.

In terms of adverse events, the pooled Odds Ratio for the incidence of any adverse events was estimated at 0.53 (95% CI = 0.06–4.82, P > 0.05), indicating that the incidence of adverse events in the HAIC group did not increase compared to the sorafenib group. According to previous related reports, the main adverse events associated with sorafenib are hand-foot skin reaction and hypertension, and the most common adverse reactions with HAIC are device-related (22–35%), followed by nausea and anorexia (28–33%), hematological toxicity (11–22%), gastritis (0–26%), and diarrhea (0–13%) [20,59]. Generally, the adverse reactions of HAIC do not overlap with those of sorafenib, suggesting that the combined application of HAIC and sorafenib for the treatment of hepatocellular carcinoma could also be a viable option. In contrast, the pooled Odds Ratio for grade 3–4 adverse event rates was 0.49 (95% CI: 0.28–0.85, P < 0.05), indicating a significantly lower incidence of severe (grade 3–4) adverse reactions with HAIC compared to sorafenib, suggesting potentially higher safety for HAIC treatment. We speculate that this may be due to several reasons. First, with advancements in technology and operational techniques, device- and technique-related complications of HAIC have significantly decreased [20]. Indeed, one study reported that only 0–4% of patients experienced catheter-related adverse events following HAIC [60]. Second, hepatic extraction of chemotherapeutic agents may result in a greater first-pass effect in the liver, minimizing systemic drug concentrations and thereby reducing systemic toxicity [61]. However, given the relatively small number of studies included in the comparison of grade 3–4 adverse events, more research may be required to validate this finding.

When interpreting the results, several limitations of this article should be taken into account: (1) The inclusion of a limited number of randomized controlled trials, with the majority being retrospective studies, may limit the generalizability and power of the findings. (2) Heterogeneity among the included studies in terms of sample characteristics and intervention protocols, such as variations in HAIC administration across different hospitals, can undermine the effectiveness of the combined analysis; To address this, we performed corresponding subgroup and sensitivity analyses to explore the specificity of the observed effects across different HAIC regimens. (3) The subjective judgments made by researchers during study selection, quality assessment, and interpretation of results are inevitable and can potentially introduce bias.

## 5. Conclusions

Among Asian and African populations, patients with BCLC stage B/C HCC treated with HAIC demonstrated higher overall survival compared to those receiving sorafenib. Moreover, among various HAIC regimens, the FOLFOX regimen conferred the greatest survival benefit. Additionally, HAIC was associated with improved oncologic outcomes, characterized by higher rates of Complete and Partial response, increased Objective response rate and Disease control rate, and a reduced likelihood of Disease progression.

## Supporting information

S1 File Search strategy. (PDF)

S2 File Quality evaluation of included studies. (XLSX)

S3 File Funnel plot of overall survival. (PDF)

S4 File Sensitivity analysis of overall survival. (PDF)

S5 File Forest plot for the comparison of SD. (PDF)

S6 File PRISMA 2020 checklist. (DOCX)

## Abbreviations

HCC: Hepatocellular carcinoma
OS: Overall survival
PFS: Progression-free survival
HAIC: Hepatic artery infusion chemotherapy
BCLC: Barcelona clinic liver cancer
CR: Complete response
PR: Partial response
SD: Stable disease
PD: Progressive disease
ORR: Objective response rate
DCR: Disease control rate
AEs: Adverse events
HR: Hazard Ratio
OR: Odds Ratio
mRECIST: the modified Response Evaluation Criteria in Solid Tumors
RECIST: the Response Evaluation Criteria in Solid Tumors
