# Supplementary material for: Hepatic arterial infusion chemotherapy (HAlC) versus sorafenib for hepatocellular carcinoma (HCC) in Barcelona Clinic Liver Cancer (BCLC) B/C: A systematic review and meta-analysis
Source: PLoS One. 2026 Feb 18;21(2):e0342495. doi: 10.1371/journal.pone.0342495 (PMC12915927; doi:10.1371/journal.pone.0342495)
Supplement: S1 File — (PDF) [file pone.0342495.s001.pdf]

S1. Search strategy

PubMed:

((Inhibitors, Tyrosine Kinase[Title/Abstract]) OR (Kinase Inhibitors, Tyrosine[Title/Abstract]) OR (Tyrosine Kinase Inhibitor[Title/Abstract]) OR (TKI Tyrosine Kinase Inhibitors[Title/Abstract]) OR (Tyrosine Protein Kinase Inhibitors[Title/Abstract])OR (sorafenib[Title/Abstract]) OR (apatinib[Title/Abstract]) OR (Lenvatinib[Title/Abstract]) OR (Cabozantinib[Title/Abstract]) OR (Regorafenib[Title/Abstract]) OR (Donafenib[Title/Abstract]) OR ("Tyrosine Kinase Inhibitors"[Mesh])) AND ((Carcinomas, Hepatocellular[Title/Abstract]) OR (Hepatocellular Carcinomas[Title/Abstract]) OR (Liver Cell Carcinoma, Adult[Title/Abstract]) OR (Liver Cancer, Adult[Title/Abstract]) OR (Adult Liver Cancer[Title/Abstract]) OR (Adult Liver Cancers[Title/Abstract]) OR (Cancer, Adult Liver[Title/Abstract]) OR (Cancers, Adult Liver[Title/Abstract]) OR (Liver Cancers, Adult[Title/Abstract]) OR (Liver Cell Carcinoma[Title/Abstract]) OR (Carcinoma, Liver Cell[Title/Abstract]) OR (Carcinomas, Liver Cell[Title/Abstract]) OR (Cell Carcinoma, Liver[Title/Abstract]) OR (Cell Carcinomas, Liver[Title/Abstract]) OR (Liver Cell Carcinomas[Title/Abstract]) OR (Hepatocellular Carcinoma[Title/Abstract]) OR (Hepatoma[Title/Abstract]) OR (Hepatomas[Title/Abstract]) OR ("Carcinoma, Hepatocellular"[Mesh])) AND ((hepatic arterial infusion chemotherapy[Title/Abstract]) OR (hepatic arterial infusion[Title/Abstract]) OR (HAIC[Title/Abstract]) OR (HAI[Title/Abstract]))

Embase:

('Inhibitors, Tyrosine Kinase':ti,ab OR 'Kinase Inhibitors, Tyrosine':ti,ab OR 'Tyrosine Kinase Inhibitor':ti,ab OR 'TKI Tyrosine Kinase Inhibitors':ti,ab OR 'Tyrosine Protein Kinase Inhibitors':ti,ab OR 'sorafenib':ti,ab OR 'apatinib':ti,ab OR 'Lenvatinib':ti,ab OR 'Cabozantinib':ti,ab OR 'Regorafenib':ti,ab OR 'Donafenib':ti,ab OR 'Tyrosine Kinase Inhibitors':ti,ab OR 'sorafenib'/exp OR 'Lenvatinib'/exp OR 'Cabozantinib'/exp OR 'Regorafenib'/exp OR 'protein tyrosine kinase inhibitor'/exp) AND ('liver cell carcinoma'/exp OR 'Carcinomas, Hepatocellular':ti,ab OR 'Hepatocellular Carcinomas':ti,ab OR 'Liver Cell Carcinoma, Adult':ti,ab OR 'Liver Cancer, Adult':ti,ab OR 'Adult Liver Cancer':ti,ab OR 'Adult Liver Cancers':ti,ab OR 'Cancer, Adult Liver':ti,ab OR 'Cancers, Adult Liver':ti,ab OR 'Liver Cancers, Adult':ti,ab OR 'Liver Cell Carcinoma':ti,ab OR 'Carcinoma, Liver Cell':ti,ab OR 'Carcinomas, Liver Cell':ti,ab OR 'Cell Carcinoma, Liver)':ti,ab OR 'Cell Carcinomas, Liver':ti,ab OR 'Liver Cell Carcinomas':ti,ab OR 'Hepatocellular Carcinoma':ti,ab OR 'Hepatoma':ti,ab OR 'Hepatomas':ti,ab OR 'Carcinoma, Hepatocellular':ti,ab) AND ('hepatic arterial infusion chemotherapy':ti,ab OR 'hepatic arterial infusion':ti,ab OR 'HAIC':ti,ab OR 'HAI':ti,ab OR 'hepatic arterial infusion chemotherapy'/exp)

Web of Science:

TS=((Inhibitors, Tyrosine Kinase) OR (Kinase Inhibitors, Tyrosine) OR (Tyrosine Kinase Inhibitor) OR (TKI Tyrosine Kinase Inhibitors) OR (Tyrosine Protein Kinase Inhibitors)OR (sorafenib) OR (apatinib) OR (Lenvatinib) OR (Cabozantinib) OR (Regorafenib) OR (Donafenib) OR (Tyrosine Kinase Inhibitors)) AND TS=((Carcinomas, Hepatocellular) OR (Hepatocellular Carcinomas) OR (Liver Cell Carcinoma, Adult) OR (Liver Cancer, Adult) OR (Adult Liver Cancer) OR (Adult Liver Cancers) OR (Cancer, Adult Liver) OR (Cancers, Adult Liver) OR (Liver Cancers, Adult) OR (Liver Cell Carcinoma) OR (Carcinoma, Liver Cell) OR (Carcinomas, Liver Cell) OR (Cell Carcinoma, Liver) OR (Cell Carcinomas, Liver) OR (Liver Cell Carcinomas) OR (Hepatocellular Carcinoma) OR (Hepatoma) OR (Hepatomas) OR (Carcinoma, Hepatocellular)) AND TS=((hepatic arterial infusion chemotherapy) OR (hepatic arterial infusion) OR (HAIC) OR (HAI))
