# Supplementary figures and images for: Hepatic arterial infusion chemotherapy (HAlC) versus sorafenib for hepatocellular carcinoma (HCC) in Barcelona Clinic Liver Cancer (BCLC) B/C: A systematic review and meta-analysis
Source: PLoS One. 2026 Feb 18;21(2):e0342495. doi: 10.1371/journal.pone.0342495 (PMC12915927; doi:10.1371/journal.pone.0342495)

Standard Error

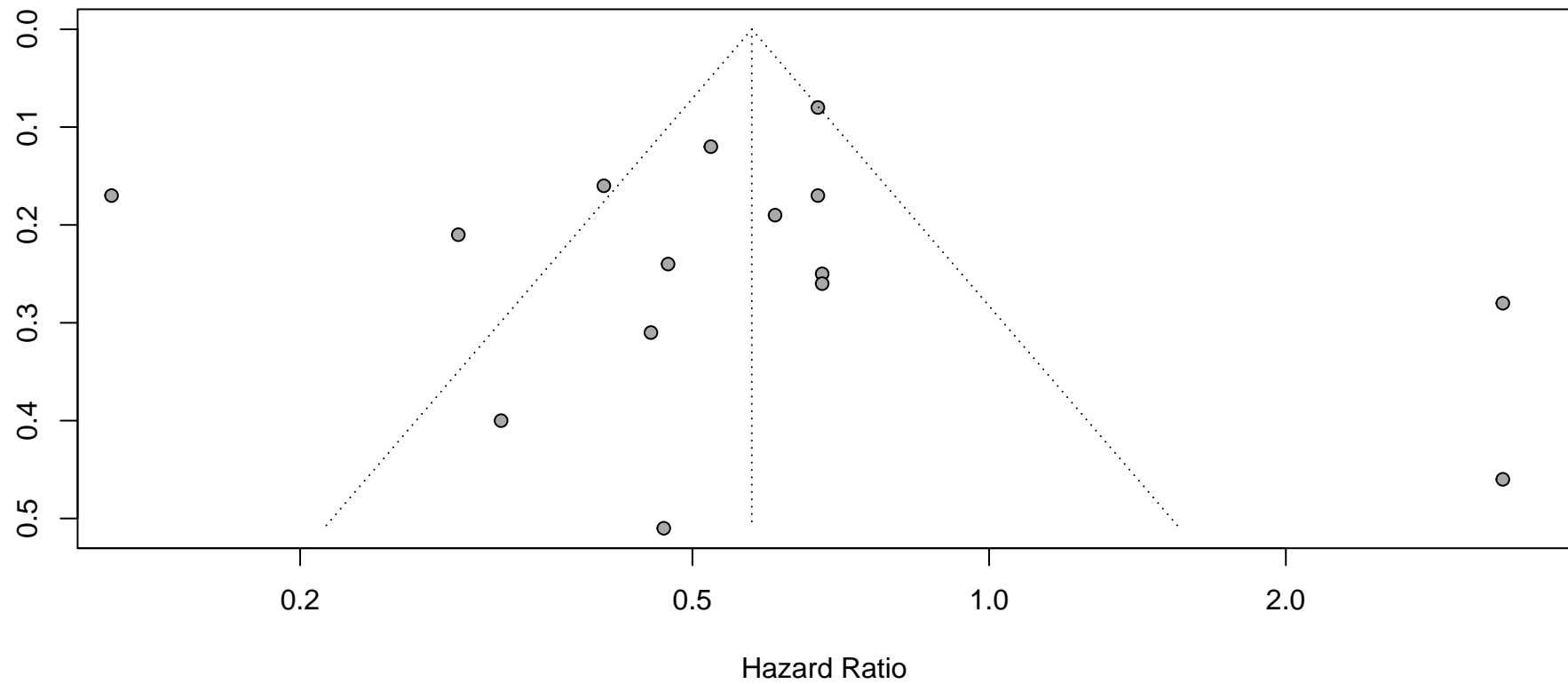

Supplement: S3 File — (PDF) [file pone.0342495.s003.pdf]

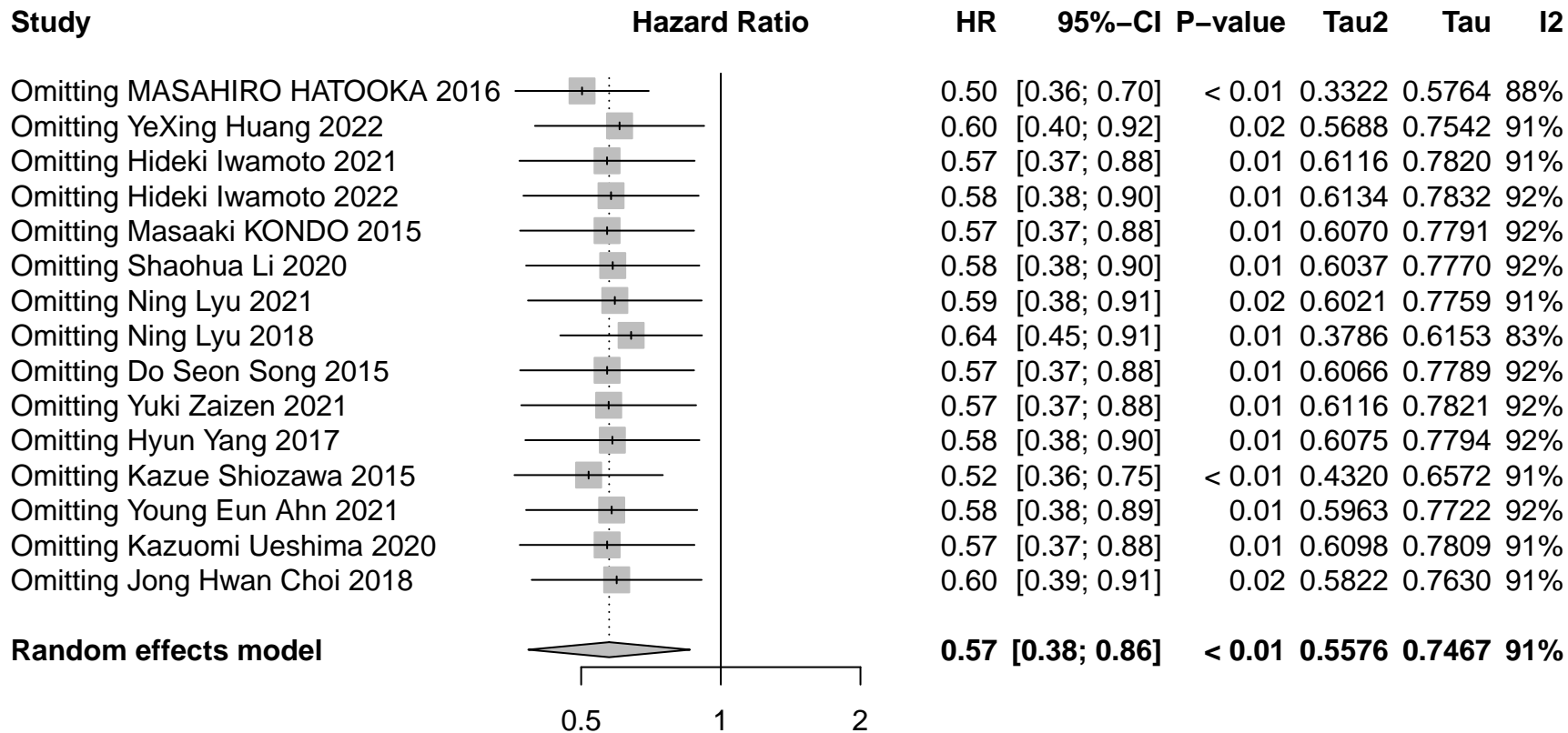

Supplement: S4 File — (PDF) [file pone.0342495.s004.pdf]

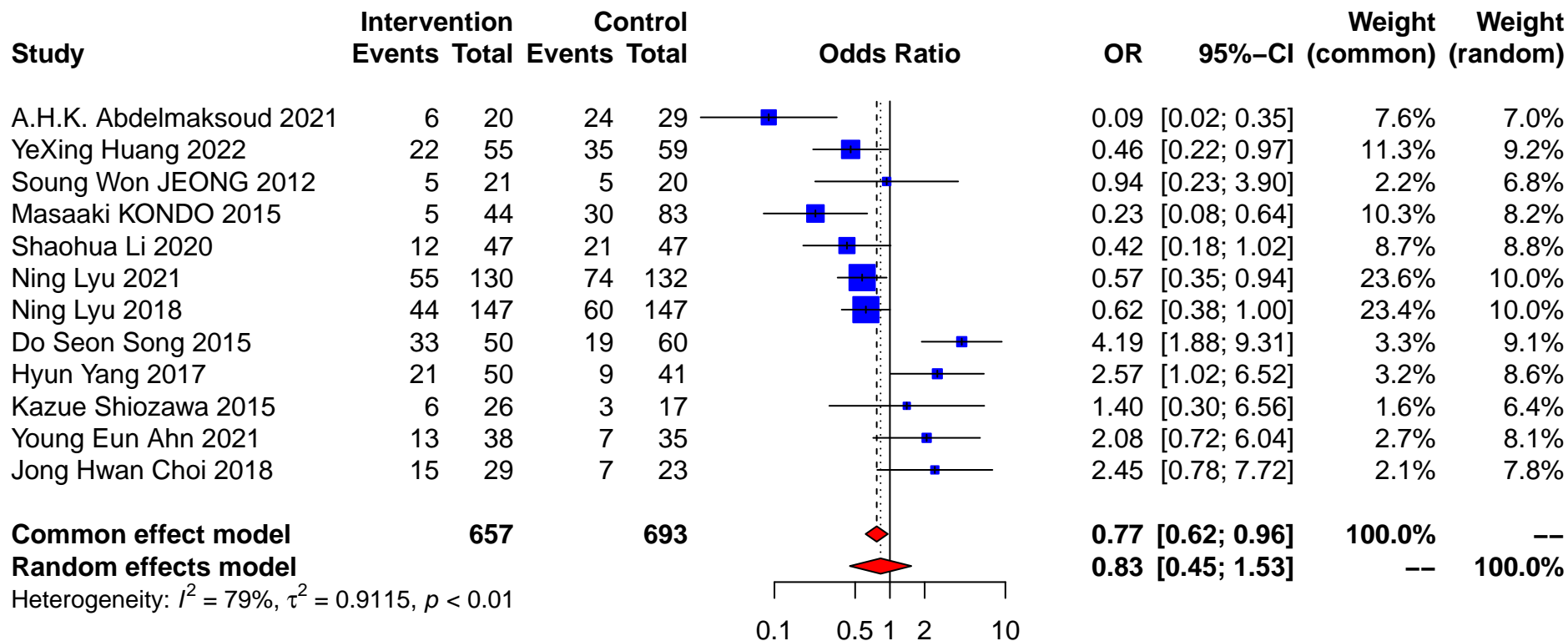

Supplement: S5 File — (PDF) [file pone.0342495.s005.pdf]
